# Hepitopes: A live interactive database of HLA class I epitopes in hepatitis B virus

**DOI:** 10.12688/wellcomeopenres.9952.1

**Published:** 2016-11-15

**Authors:** Sheila Lumley, Howard Noble, Martin J. Hadley, Liz Callow, Amna Malik, Yi Yi Chua, Owen J. Duffey, Natalia Grolmusova, Arvind Kumar, Samuel Ravenscroft, Jonathan I. Spencer, Christoph Neumann-Haefelin, Robert Thimme, Monique Andersson, Paul Klenerman, Eleanor Barnes, Philippa C. Matthews

**Affiliations:** 1 Department of Infectious Diseases and Microbiology, Oxford University Hospitals NHS Foundation Trust, John Radcliffe Hospital, Oxford, UK; 2 IT Services, University of Oxford, Oxford, UK; 3 Bodleian Health Care Libraries, University of Oxford, John Radcliffe Hospital, Oxford, UK; 4 Department of Paediatrics, University of Oxford, Oxford, UK; 5 University of Oxford Medical School, John Radcliffe Hospital, Oxford, UK; 6 Department of Medicine II, University Hospital Freiburg, Freiburg, Germany; 7 Nuffield Department of Experimental Medicine, University of Oxford, Oxford, UK; 8 NIHR Biomedical Research Centre, John Radcliffe Hospital, Oxford, UK

**Keywords:** Hepatitis B Virus, HBV, epitope, CD8+ T cell, HLA class I, database

## Abstract

Increased clinical and scientific scrutiny is being applied to hepatitis B virus (HBV), with focus on the development of new therapeutic approaches, ultimately aiming for cure. Defining the optimum natural CD8+ T cell immune responses that arise in HBV, mediated by HLA class I epitope presentation, may help to inform novel immunotherapeutic strategies. Therefore, we have set out to develop a comprehensive database of these epitopes in HBV, coined ‘Hepitopes’. This undertaking has its foundations in a systematic literature review to identify the sites and sequences of all published class I epitopes in HBV. We also collected information regarding the methods used to define each epitope, and any reported associations between an immune response to this epitope and disease outcome. The results of this search have been collated into a new open-access interactive database that is available at http://www.expmedndm.ox.ac.uk/hepitopes. Over time, we will continue to refine and update this resource, as well as inviting contributions from others in the field to support its development. This unique new database is an important foundation for ongoing investigations into the nature and impact of the CD8+ T cell response to HBV.

## Introduction

Hepatitis B virus (HBV) is the prototype virus in the *Hepadnaviridae* family. It has unique features that make it interesting and distinct from other related viruses. Its unusual, partially double-stranded circular genomic structure, comprising several overlapping reading frames ^1^, represents a potential barrier to variability – any nonsynonymous nucleic acid substitution potentially has to be tolerated in the resulting sequences of more than one protein product ^2^. However, its error-prone reverse transcriptase enzyme, responsible for the generation of nucleic acid intermediates, is a source of diversity that is unusual in a DNA virus ^3– 5^.

HBV is estimated to infect 240 million people globally, and to cause over half a million deaths each year ( http://www.who.int/mediacentre/factsheets/fs204/en/). At present, therapy consists of peginterferon alfa-2a, tenofovir disoproxil or entecavir ( https://www.nice.org.uk/guidance/cg165). Although successful in mediating viral suppression, these drugs, either alone or in combination, do not commonly mediate cure, and many patients are committed to life-long therapy. However, the striking recent success of direct acting antiviral therapy for HCV has sparked new enthusiasm for the vision of a HBV cure ^6^.

To optimize our chances of HBV eradication, there are a number of important public health interventions that must be pursued in parallel, including diagnostics, treatment and prevention. A crucial component of this strategy is the development and testing of new drug agents, and the investigation of immunotherapeutic interventions ^7, 8^. There is growing interest in the idea of a therapeutic vaccine that could boost or mimic the immune responses that correlate best with HBV clearance in natural infection ^6, 9, 10^.

There are several strands of evidence for an important role of the CD8+ T cell response in HBV. Associations have been documented between HLA genotype and disease outcome ^11– 13^, including responses to, and recovery from, acute infection ^14– 16^. In patients that clear HBV infection, interferon (IFN)-gamma producing CD8+ T cells are enriched, compared to those with chronic infections ^17^. The recent literature demonstrates an emerging interest into investigations of this CD8+ mediated influence on HBV, including the identification of HLA class I epitopes (for examples, see references ^13, 18, 19^; for a full dataset, see ‘Data availability’ section), and reports of HLA-driven selection pressure driving viral diversity ^20, 21^.

This dynamic between host and virus is well described for HIV ^22– 24^, in which the HLA class I restricted T cell response is a major influence on disease control ^25– 27^. An on-line database of HIV epitopes ( https://www.hiv.lanl.gov/content/immunology/ctl_search) is an important resource in pooling knowledge and resources, and driving collaboration, in the field of HIV T cell immunology. Although there are several useful on-line repositories for HBV sequence data (including HBV-specific databases https://hbvdb.ibcp.fr/HBVdb/ ^2^ and http://www.hepseq.org, as well as HBV sequences that can be found in broader sequence repositories, such as http://www.ncbi.nlm.nih.gov), and a recent publication collates the most robustly reported HBV class I epitopes ^28^, there is no current publically-available database of HBV epitopes to parallel the successful resource that has grown and developed for HIV ( https://www.hiv.lanl.gov/content/immunology/ctl_search).

Therefore, we have begun the process of collating HBV class I epitopes. Our primary aim was to provide a detailed and comprehensive database of epitopes that have been identified to date, but with an ongoing remit to develop this into a sustainable open-access online research resource that evolves over time.

## Materials and methods

### Systematic literature review

We searched two online bibliographic databases, Medline and Embase, in January 2016, using the OVID search interface ( http://ovidsp.ovid.com/) made available by the University of Oxford. No date restrictions were imposed; Medline was searched from 1946–2016 and Embase from 1974–2016. The relevant subject headings for Epitopes, HLA Antigens, CD8 Antigens and Hepatitis B from the thesauri (MESH and EMTREE) were exploded and searched. In addition, the terms ‘epitope*’ (to pick up singular and plural) and ‘Hepatitis B’ and ‘HBV’ were searched in the title and abstract fields. The terms of the search strategy and the Boolean operators used are presented in Table 1. We also identified three additional references by searching the bibliographies of articles that had been identified in the primary search. These can be identified within our dataset (by searching the column entitled ‘database’ which specifies the origin of the citation).

**Table 1.** Search strategy for systematic literature review to identify HLA class I epitopes in hepatitis B virus (HBV). This table represents the two databases searched by our literature review, the dates of publication included in the search, and the search terms used to identify relevant citations. ‘Exp’ denotes that the term has been selected from the database thesaurus and exploded to pick up its narrower terms. The / sign denotes that the term is a subject heading from the thesaurus. ‘Epitope*’ includes search for ‘epitope’ and ‘epitopes’. ‘.mp’ denotes a search of the title, abstract, original title, name of substance word, subject heading word, keyword heading word, protocol supplementary concept word, rare disease supplementary concept word, and unique identifier.

Database	Embase	Medline
**Date searched**	January 1974 – January 2016	January 1946 – January 2016
**Search strategy**	1. exp epitope/ 2. epitope*.mp 3. 1 or 2 4. exp HLA antigen/ 5. exp CD8 antigen/ 6. 4 or 5 7. exp Hepatitis B/ 8. (Hepatitis B or HBV).mp. 9. 7 or 8 10. 3 and 6 and 9	1. exp epitopes/ 2. epitope*.mp 3. 1 or 2 4. exp HLA Antigens/ 5. exp Antigens, CD8/ 6. 4 or 5 7. exp Hepatitis B/ 8. (Hepatitis B or HBV).mp. 9. 7 or 8 10. 3 and 6 and 9

Using this approach, we identified 447 papers in total. We reviewed each paper to ensure it met our search criteria, excluding duplicates (n=113), those not relevant or missing essential data (n=177), publications not written in English (n=26) and those that we were unable to access (n=19), leading to the inclusion of final data from 112 papers. From these, we recorded the following details: citation, epitope location and sequence, HLA restriction, experimental approach used to confirm the epitope, and any documented association with stage of HBV disease. From the selected 112 manuscripts, we collated a database of HBV epitopes using Google Sheets ( https://docs.google.com/spreadsheets) each of which has documented optimal and/or overlapping peptide sequence and defined HLA-class I restriction.

### Sequence numbering

In order to unify our approach to numbering amino acid position within HBV proteins, we used a reference sequence published by Liu *et al*. ^29^. In order to support consistent alignment, and to identify potential genotype-specific differences that may affect epitope presentation or recognition, we have also defined consensus sequences for each protein based on all available sequences downloaded from https://hbvdb.ibcp.fr/HBVdb/ ^2^. The reference sequence and aligned genotype-specific consensus sequences are available on-line (DOI: http://dx.doi.org/10.6084/m9.figshare.4040700.v1 ^30^).

### Interactive database

**Figure 1.**
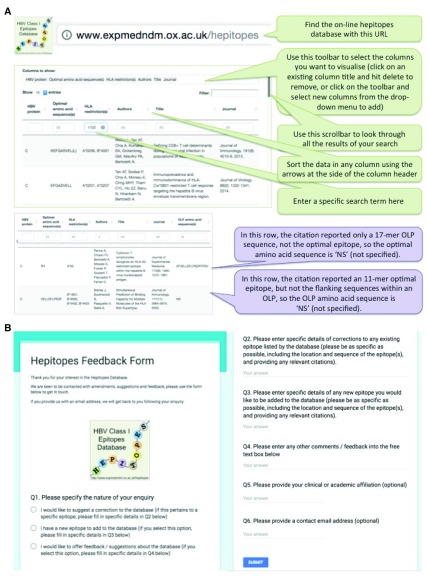
Screenshots of the Hepitopes database ( http://www.expmedndm.ox.ac.uk/hepitopes). ( **A**) Appearance of the data visualization on-line, with notes on interrogation of the database (green boxes) and features of the output (purple boxes). ( **B**) On-line contact portal to develop dialogue and collaborations and to feed updates into the database.

The results of the literature search, which will form the basis of an expanding data resource, can be viewed at http://www.expmedndm.ox.ac.uk/hepitopes as an interactive web element to facilitate other researchers in exploring and querying the dataset ( Figure 1A). This web element utilizes the widely used JavaScript library Datatables (SpryMedia Limited v. 1.10.12; datatables.net) to allow the data to be filtered and sorted interactively. Hosting is provided by RStudio webservice (shinyapps.io) and the web element was developed using the R framework Shiny (Shiny: Web Application Framework for R, v. 0.14.1.; https://CRAN.R-project.org/package=shiny) ^31^.

The interactive web element was developed as a case study of the Live Data project led by Research Support Services and funded by IT Services at University of Oxford. The project was initiated to investigate whether a central interactive visualization service would increase engagement with datasets underlying research projects, and foster additional research impact. Hepitopes provided a template case study for researchers interested in making creating interactive interfaces to databases and flat files.

We will continue to curate the Hepitopes on-line database, adding amendments and updates over time. To facilitate wider contributions from the scientific community, our website includes a contact portal ( Figure 1B); we welcome contributions of new data and citations, corrections to existing information, or general feedback about the site and resource, with the aims of refining the quality of the dataset, encouraging dialogue and collaboration, and adding new tools, including links to other relevant resources.

## Dataset validation

The results of our primary literature search are limited by our ability to identify all reports that may contain pertinent data; it may be that we have overlooked relevant citations due to the data being presented in manuscripts that did not contain our specific search terms. Furthermore, we were unable to access the complete report for 19 citations, which may contain relevant data (e.g. conference abstracts, for which the entire dataset has not been published electronically). We recognize that the nature and quality of the data presented in different reports is heterogeneous, due to different genotypes of HBV studied presented with varying alignments, and a diverse range of methodological approaches ranging from *in silico* prediction to detailed *in vitro* elucidation of epitopes in natural infection or in animal models.

It is important to recognize that the predominance of any particular HLA class I molecule may not reflect a true biological immunodominance hierarchy, but rather is a result of the bias towards identification, reporting and further investigation of epitopes restricted by the HLA class I alleles that occur at the highest phenotypic frequency in the majority of human populations. This is illustrated by HLA-A*02 epitopes, which - at the point of releasing the database - account for 42% of the total and are referenced in 107 of the cited manuscripts.

We also acknowledge that the HBV literature is skewed towards investigation of certain populations, most specifically in the Far East, where HBV genotypes B and C are endemic ^32^, and potentially also in Western Europe and North America where better resources are available for these studies. On these grounds, the results of our literature search over-represent certain host/virus interactions, specifically between HBV genotype-B/C and host HLA class I alleles represented at the highest phenotypic frequency in these populations. An alternative source of bias in the existing data is the peptide sets used for *in vitro* confirmation of optimal epitopes, among which genotype D is anecdotally over-represented (although this methodological bias is difficult to quantify in any systematic way). The aim is that, as the resource develops over time, we will build a more comprehensive picture of the true spectrum of HLA class I presented epitopes, and gather data from populations that have been under-represented to date, including our own particular focus of interest in southern Africa ^33, 34^.

## Data and software availability

### On-line visualization of the Hepitopes database

The live interactive database containing the collated results of our literature review is available via the Hepitopes project website: http://www.expmedndm.ox.ac.uk/hepitopes. This visualisation is represented by the screenshots in Figure 1, together with some notes about use of this interface. The code for interactive elements is made available under an MIT license and is hosted on GitHub, https://github.com/ox-it/hepitopes (archived version at the time of publication: DOI: http://dx.doi.org/10.5281/zenodo.163106 ^35^).

### Raw data

Data is ingested into the web element via a Figshare repository (DOI: http://dx.doi.org/10.6084/m9.figshare.4015824 ^36^), which we will continue to update over time. Additionally, a snapshot of the data at the time of submission has been deposited on the Oxford Research Archive (ORA; DOI: http://dx.doi.org/10.5287/bodleian:zr0VAr78q ^37^; https://ora.ox.ac.uk/objects/uuid:9aa65287-5027-4422-abd1-f697aa3f7dc9).

### Aligned reference and consensus sequences for HBV proteins

Our sequence alignment, including a description for the way this was generated, is available from Figshare (DOI: http://dx.doi.org/10.6084/m9.figshare.4040700.v1 ^30^).

### Access to citations represented in the Hepitopes database

The DOI (digital object identifier, issued for articles published after the year 2000) listed for each citation within the Hepitopes database is in the public domain. Access to the abstract and full text is subject to institutional or individual subscription to the journal, or open access availability.

## Ethics

Patient consent was not required for this project, and no specific ethics approval was required. The original manuscripts cited in our database should be consulted individually for details of specific ethics approvals and consent, if required.
