# Supplementary material for: Hepitopes: A live interactive database of HLA class I epitopes in hepatitis B virus
Source: Wellcome Open Res. 2016 Nov 15;1:9. [Version 1] doi: 10.12688/wellcomeopenres.9952.1 (PMC5142601; doi:10.12688/wellcomeopenres.9952.1)
Supplement: Peer review history

# Peer review: Referee response for version 1

**DOI:** 10.21956/wellcomeopenres.10727.r17597

**Published:** 2016-12-05

**Authors:** Joerg Timm

**Affiliations:** 1 Institute of Virology, Heine-University, University Hospital, Düsseldorf, Germany

The authors describe a novel and publically available database of CD8+ T cell epitopes in HBV. Our knowledge of the targeted HBV epitopes is still limited and until now no comprehensive collection of all described epitopes is available. The database is therefore of great interest to researchers working in the field of HBV T cell immunology and potentially is an important tool for the design of therapeutic vaccination strategies.

 The manuscript is well written and gives all relevant details about the strategy of data mining from the literature. The title is appropriate and the abstract provides an adequate summary of the article. Although the database is of great value, there are a few limitations that need to be addressed.

 I have the following comments:

- It is unclear to me how the search criteria were selected. It seems to me that epitopes from relevant publications have not been included in the database (e.g. doi: 10.1002/hep.27771), despite having been mentioned in the manuscript. I understand that the existing data have not yet been validated. But the authors should reconsider the search strategy to include more epitopes.
- The authors state that they will continue to curate the database in the future. As I understand, this will be done based on information provided by users and by additional literature searches. In line with my first comment, the literature search should be broadened in order to avoid missing relevant publications. More importantly, will there be a quality check of entries provided by users? If so, the authors should describe how this is done.
- I believe, the database would be more helpful, if the epitopes were listed as a single entry with all relevant references linked to it. Some epitopes have been described in multiple publications (e.g. the A*02-restricted core epitope FLPSDFFPSV is listed 56 times). This complicates the search for individual epitopes.
- I believe, it would strongly help if additional information about the assay used for identification of the epitope was included in the database. It is quite different if an epitope was experimentally confirmed by a functional assay or HLA class I-multimers, if HLA class I-peptide-binding was determined or if the epitope was only predicted *in silico*.
- Do the authors consider including CD4+ T cell epitopes as well? Is there a rational why the database is limited to CD8+ T cell epitopes?

I have read this submission. I believe that I have an appropriate level of expertise to confirm that it is of an acceptable scientific standard.

# Peer review: Referee response for version 1

**DOI:** 10.21956/wellcomeopenres.10727.r17596

**Published:** 2016-12-01

**Authors:** Louise Corti

**Affiliations:** 1 UK Data Archive, University of Essex, Colchester, UK

**Cataloguing efforts**

 This retrospective collation and cataloguing work of sites and sequences of all published class I epitopes in HBV (Hepitopes) is a useful contribution to the knowledge base in this field. This systematic literature review has clearly been a heavily manual effort as information is so dispersed. The two online bibliographic databases that were selected to undertake targeted searches, Medline and Embase, may not offer 100% coverage and what about other resources in other languages? The ‘universe’ is not explicitly mentioned in the paper and needs to be added.

**Technology and sustainability**

 I found the website easy to use, but at times, unstable, with multiple crashes on the site/ disconnection from the server. It is good to see the use of open source tools, for example, Shiny R. The interactivity is quite basic, based on column sorting (effectively an online spreadsheet) but nevertheless useful. I found the feedback and contributor process to be manual and quite primitive, through what is essentially a minimally structured paper-based form. It would nice to see a more automated upload process with structured fields available for population and validation on the fly; and some local vetting once an entry is submitted.

 In terms of future proofing and keeping data up to date, the team note that they have an ongoing remit to develop this into a sustainable open-access online research resource that evolves over time. While the code is usefully for the datatable available on Github, it is not that clear how any maintenance of the project might be supported and under what budget. The team have promised to continue to refine and update this resource, yet what happened if key staff leave?

 Future proofing is the main concern of so many local resources like this, which is why the Research Council’s typically try not to fund on-going longer-term information resources under the auspices of smaller one-off research projects due to the lack of future accessibility. ESRC have set up a centralised web based ReStore to ensure that their web-based research methods resources created under one-time research awards are maintained in the longer term – either as maintained resources or static snaphot web-based resources. So may resources created with the very best intentions, are not kept up to date and may become out of date quickly, and this become a relic

 While I really embrace the idea of crowd sourcing information to maintain future, in my opinion the physical resource /database (the catalogue) should be looked after by an official long standing trusted repository e.g. Genbank and not an individual university. The fact that the authors mention another almost identical on-line database of epitopes, but for HIV (( https://www.hiv.lanl.gov/content/immunology/ctl_search), and other online repositories in the field of HIV T cell immunology (sequence data including HBV-specific databases https://hbvdb.ibcp.fr/HBVdb/ 2 and http://www.hepseq.org, and in broader sequence repositories, such as http://www.ncbi.nlm.nih.gov) makes me wonder why a unified database has not been proposed with a single interface! The last thing the community needs is a proliferation of locally managed lists so why not combine them into one infrastructure? This professionalised future proofing of resources like this really needs to be addressed in the paper, even if they are aspirational.

 Finally, if the web resource/front end was not to be retained in the future this snapshot database is usefully backed up in Figshare. The paper says the team will keep this updated, but it is not clear how future dumps of the dataset might be formally archived, persistently cited, and referenced in Figshare and how formal versioning might work. Will Oxford and Fighare sync their versions? This area needs to be addressed to make it clear they have really considered future plans for this useful table of information.

I have read this submission. I believe that I have an appropriate level of expertise to confirm that it is of an acceptable scientific standard, however I have significant reservations, as outlined above.

# Peer review: Referee response for version 1

**DOI:** 10.21956/wellcomeopenres.10727.r17603

**Published:** 2016-11-24

**Authors:** Antonio Bertoletti, Anthony Tanoto Tan, Upkar S Gill

**Affiliations:** 1 Emerging Infectious Diseases Program, Duke-National University of Singapore Graduate Medical School, Singapore, Singapore; 2 Emerging Infectious Diseases Research Program, Duke-National University of Singapore Graduate Medical School, Singapore, Singapore; 3 Hepatology, Centre for Immunobiology, Blizard Institute, Barts and The London School of Medicine & Dentistry, Queen Mary University of London, London, UK

This manuscript describes the creation of a database cataloging epitopes derived solely from Hepatitis B virus. The authors need to be congratulated for their effort to set up a clear and user friendly resource that provides all the current scientific information known on HBV epitopes at present. 

 What I found particularly nice is that it details how the different epitopes were experimentally determined and this allow the user to understand whether the epitope was able to induce a T cell response in a subject with natural infection or was only the result of *in silico* determination and binding assays.

 Such a resource will allow easy progression of future research in T cell immunology. Researchers will have access to the information on relevant HBV epitopes in one dataset, which will be both convenient and time-saving. In addition as noted, the experimental technique of epitope determination is noted which can further direct experimental design.

 As noted by the authors, the resource, will hopefully, can continue to grow, certainly  considering the number of people who are screening HBV patients, this might not be a trivial task. In addition it is also of value that comments from readers can be added and thus allow authors to update the resource as appropriate. Thus the importance being that the resource will evolve over time and therefore be an invaluable asset in HBV research.

 The authors of the manuscript/resource have clearly outlined their methods for the literature search in developing the interactive database which are robust. I also note the limitations of dataset validation, whereby citations could be overlooked as they are not search specific. However, accepting this I have a couple of suggestions, that I hope can be useful to improve the utility of the site:

  

1. The data gathered do not appear to be consolidated and as such do not generate a “final” state of knowledge on HBV epitopes landscape. The website provides all the information but they are listed as a list of papers. This can create potential confusion, since for example if you are interested to know whether the core 18-27 FLPSDFFPSV epitope comprises a polymorphism (which it does) you need to ask this information when you are reading the relevant paper, where such polymorphism was demonstrated. If you are asking this identical question starting from a paper where such polymorphism was not analyzed, the answer is that there is no polymorphism in this region. I think this is an aspect that could be improved since it might generate some misinterpretation. 
2. A similar problem is also present in relation to the impact of HLA-A02 subtypes. Some epitopes are presented as pan A2 based on old papers but more recent publication  (Tan AT *et al.* 2008) have show that some epitopes are exclusively A0201. For example the envelope epitopes FLLTRILTI or FLLTKILTI were demonstrated exclusively on HLA-A0201 subjects and not on HLA-A0203, 0206, 0207 subjects.) The progressive evolution of information is somehow lost. Thus at places where the term ‘pan’ is used this may need to be verified as new data may not corroborate this. Having said that I appreciate the limitations, as noted in the manuscript regarding data validation. 
3. When asking for a list of, for example, HBV epitopes within the core region, the web site provides all the papers where core epitopes were demonstrated but there is a lot of redundancy. There are 6 pages listing all the papers published using the core 18-27 epitope. I am wondering whether there could be a method to first list all the different epitopes with the indication of the number of publications where such epitope was reported as a measure of the evidence supporting the validity of the epitope. This may be a method to improve the resource.
4. The description given in the database for the method of epitope characterization is very detailed, but what may not immediately be clear is whether the method used is accurate enough to give you real HBV epitopes.  For example, if the epitopes were characterized using the T cells from naturally infected patients and targets where HBV proteins were endogenously processed, that would be ideal. If the epitope was only characterized by predictive algorithms, then it would be less so. Perhaps a scale (Patient T cells with endogenously processed epitopes > Patient T cells with peptide pulsed targets > binding assays > predictive algorithms) indicating the validity of the consolidated epitopes might be clearer for the users of the database. 
5. As some citations may have been missed due to the search criteria, it may be a possibility to widen the search. This may be more feasible once readers have provided feedback. This will allow the search to be increased possibly to include all the relevant data for the resource.

We have read this submission. We believe that we have an appropriate level of expertise to confirm that it is of an acceptable scientific standard.

## References

[1] TanATLoggiEBoniCChiaAGehringAJSastryKSGohVFisicaroPAndreonePBranderCLimSGFerrariCBihlFBertolettiA: Host ethnicity and virus genotype shape the hepatitis B virus-specific T-cell repertoire. *J Virol*.2008;82(22) : 10.1128/JVI.01124-08 10986-97 10.1128/JVI.01124-08 18799575PMC2573267
